# pH-Responsive Non-Ionic Diblock Copolymers: Ionization of Carboxylic Acid End-Groups Induces an Order–Order Morphological Transition**

**DOI:** 10.1002/anie.201409799

**Published:** 2014-11-21

**Authors:** Joseph R Lovett, Nicholas J Warren, Liam P D Ratcliffe, Marzena K Kocik, Steven P Armes

**Affiliations:** Dainton Building, Department of Chemistry, The University of SheffieldSheffield, South Yorkshire, S3 7HF (UK)

**Keywords:** block copolymers, morphological transitions, nanoparticles, RAFT polymerization, self-assembly

## Abstract

A carboxylic acid based reversible additionfragmentation transfer (RAFT) agent is used to prepare gels composed of worm-like diblock copolymers using two non-ionic monomers, glycerol monomethacrylate (GMA) and 2-hydroxypropyl methacrylate (HPMA). Ionization of the carboxylic acid end-group on the PGMA stabilizer block induces a worm-to-sphere transition, which in turn causes immediate degelation. This morphological transition is fully reversible as determined by TEM and rheology studies and occurs because of a subtle change in the packing parameter for the copolymer chains. A control experiment where the methyl ester derivative of the RAFT agent is used to prepare the same diblock copolymer confirms that no pH-responsive behavior occurs in this case. This end-group ionization approach is important for the design of new pH-responsive copolymer nano-objects as, unlike polyacids or polybases, only a minimal amount of added base (or acid) is required to drive the morphological transition.

There has been substantial and sustained interest in the field of stimulus-responsive polymers over the last two decades.[1] Two particularly well-studied stimuli are thermal[1b,c] and pH[1d,e] triggers for water-soluble polymers, which can be exploited for various biological applications.[2] Of particular relevance to the present work, various research groups have exploited end-group effects to induce either self-assembly or morphological transitions.[3] For example, Stöver et al. have reported that the lower critical solution temperature (LCST) of poly(*N*-isopropylacrylamide) (PNIPAM) can be tuned over a wide range by introducing hydrophilic or hydrophobic end-groups, respectively.[3a] Similarly, Perrier, Warr, and co-workers found that the aqueous solution behavior of a series of non-ionic polymeric surfactants based on PNIPAM oligomers depended critically on whether a terminal carboxylic acid group was ionized or not.[3b] Furthermore, Gibson and co-workers utilized hydrophilic disulfide linkages to increase the LCST of PNIPAM: it proved possible to trigger a coil-to-globule collapse at a constant temperature simply by reduction of the hydrophilic end-group using glutathione.[3c] In related work, Rimmer et al. described the reversible addition–fragmentation transfer (RAFT) synthesis of highly branched fluorescently labeled PNIPAM and showed that its interaction with certain bacteria triggered a coil-to-globule transition, which in principle could be exploited as a microorganism sensor.[3d] O’Reilly and Moughton used a quaternary amine-functionalized RAFT chain transfer agent (CTA) to prepare a PNIPAM-based diblock copolymer, which self-assembled to form spheres at room temperature.[3e] However, heating above the LCST of the PNIPAM stabilizer altered the packing parameter and induced a morphological sphere-to-vesicle transition: the cationic charge conferred by the CTA-derived end-group located on the PNIPAM chains ensured colloidal stability, rather than macroscopic precipitation. Weaver et al. found that the water solubility of a series of near-monodisperse poly(2-hydroxyethyl methacrylate) homopolymers prepared using atom transfer radical polymerization (ATRP) critically depended on the solution pH because of the *N*-morpholine-based initiator used for their synthesis.[3f] Very recently, Du and co-workers reported that a terminal alkynyl end-group was capable of driving the self-assembly of hydrophilic PNIPAM and poly(oligo(ethylene glycol) methacrylate) homopolymers to form various morphologies in aqueous solution.[3g]

Amphiphilic diblock copolymers undergo self-assembly in water to form various nano-objects, such as spherical micelles, cylindrical micelles (e.g. rods or worms), or vesicles.[4] Varying the relative volume fractions of each block usually dictates the final copolymer morphology, although kinetically trapped morphologies are quite common.[4b,c] In principle, such nano-objects can be utilized for drug delivery, microencapsulation, and catalysis.[5] Normally, self-assembly requires post-polymerization processing using a pH or solvent switch, which is invariably conducted in dilute aqueous solution (<1 wt. %).[6]

Recently, we have shown that polymerization-induced self-assembly (PISA) can be utilized to prepare a wide range of nano-objects at relatively high concentration directly in aqueous solution.[7] For example, we reported chain extension of poly(glycerol monomethacrylate) (PGMA) macro-CTA using 2-hydroxypropyl methacrylate (HPMA) through RAFT aqueous dispersion polymerization.[8] For a relatively narrow range of target diblock compositions and copolymer concentrations, a pure worm-like phase can be reproducibly obtained.[8c] Such PGMA–PHPMA worm-like structures form soft transparent free-standing gels at 20 °C. However, a worm-to-sphere morphological transition occurs upon cooling to 5 °C, which leads to rapid degelation.[8d, 9] This reversible transition enables facile worm gel sterilization through cold ultrafiltration of the low-viscosity spherical phase, which suggests biological applications for these biocompatible hydrogels. Herein, we report that such non-ionic PGMA–PHPMA diblock copolymer worms can also unexpectedly exhibit pH-responsive character. We believe that this discovery offers considerable scope for the design of new stimulus-responsive polymers.

A PGMA_56_ macro-CTA containing a terminal carboxylic acid group was prepared by RAFT solution polymerization in ethanol using 4-cyano-4-(2-phenylethane sulfanylthiocarbonyl) sulfanylpentanoic acid (PETTC) as a chain transfer agent (see Figure 1 a). This near-monodisperse water-soluble macro-CTA was then chain-extended using RAFT aqueous dispersion polymerization of HPMA at 70 °C at approximately pH 3.5. ^1^H NMR spectroscopy confirmed that more than 99 % HPMA conversion was achieved at 10 % copolymer concentration. Gel permeation chromatography (GPC) in dimethylformamide (DMF) indicated a relatively high chain extension efficiency and a low copolymer polydispersity (*M*_w_/*M*_n_<1.20). TEM studies indicated that the resulting diblock copolymer worms had a well-defined mean width of 21 nm, while the worm contour length was less well-controlled and ranged from 200 to 850 nm. The resulting HOOC-PGMA_56_–PHPMA_155_ worms form a soft, transparent gel at 10 % w/w copolymer concentration in mildly acidic solution (pH<4) as a result of multiple contacts between the individual worms. Degelation occurs rapidly upon cooling this gel, because, according to Blanazs et al.,[8c] the polymer worms are transformed into spheres. This transformation occurs as a result of the greater hydration of the core-forming PHPMA block, since this reduces the overall packing parameter, *P*, for the copolymer chains.[4c] However, such non-ionic PGMA–PHPMA diblock copolymer worms also exhibit pH-responsive behavior, with degelation being observed on increasing the solution pH from pH 3.5 to 6.0 using NaOH (see Figure 1 b, and Figure 2 a and b). Furthermore, returning the solution pH to its original value resulted in reformation of the worms and thus regelation of the aqueous solution (see Figure 2 c and d). This reversible behavior suggested that in situ chemical degradation of the copolymer was unlikely.

**Figure 1:**
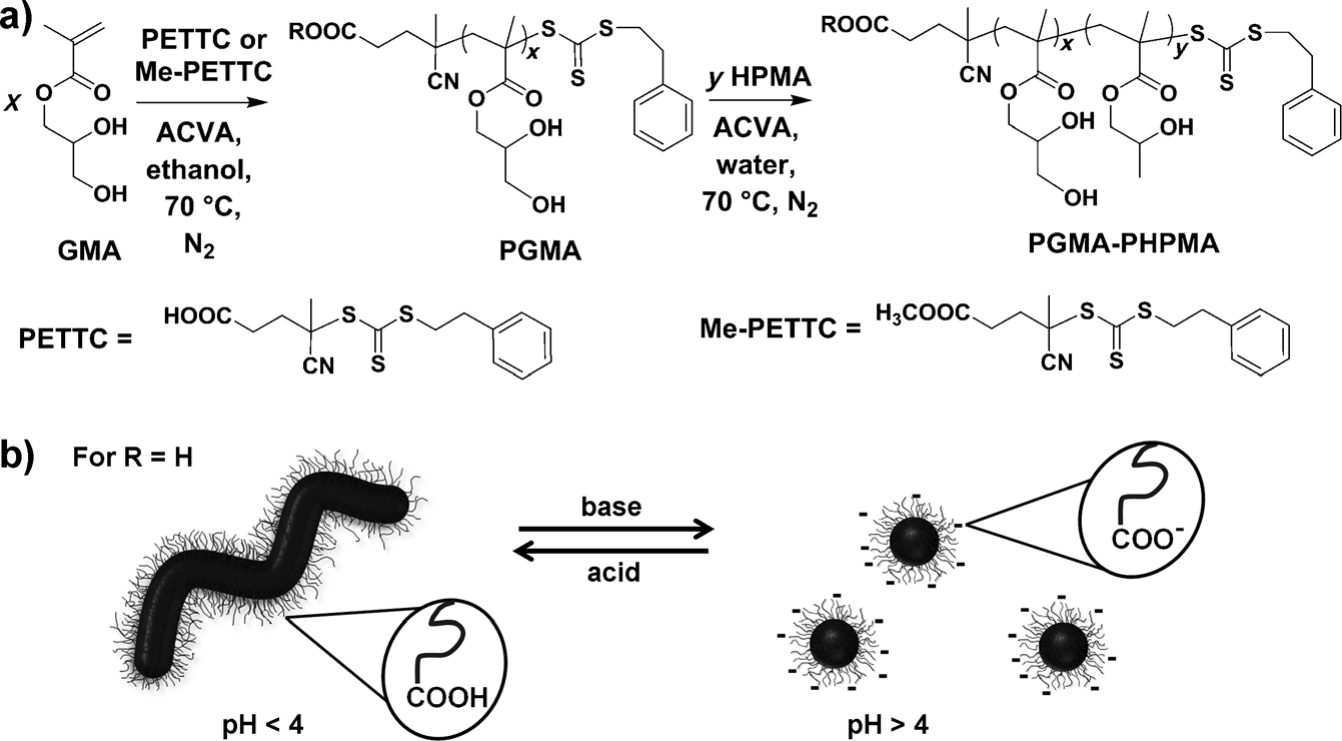
a) Synthesis of a PGMA_56_ macro-CTA by RAFT solution polymerization using a 4-cyano-4-(2-phenylethane sulfanylthiocarbonyl) sulfanylpentanoic acid (PETTC) RAFT agent, and its subsequent chain extension with HPMA to form PGMA–PHPMA diblock copolymer nano-objects at pH 3.5. b) Illustration of the worm-to-sphere morphological transition when COOH-functionalized worms are subjected to a pH change upon addition of base. ACVA= 4,4′-azobis(4-cyanopentanoic acid), the radical initiator.

**Figure 2:**
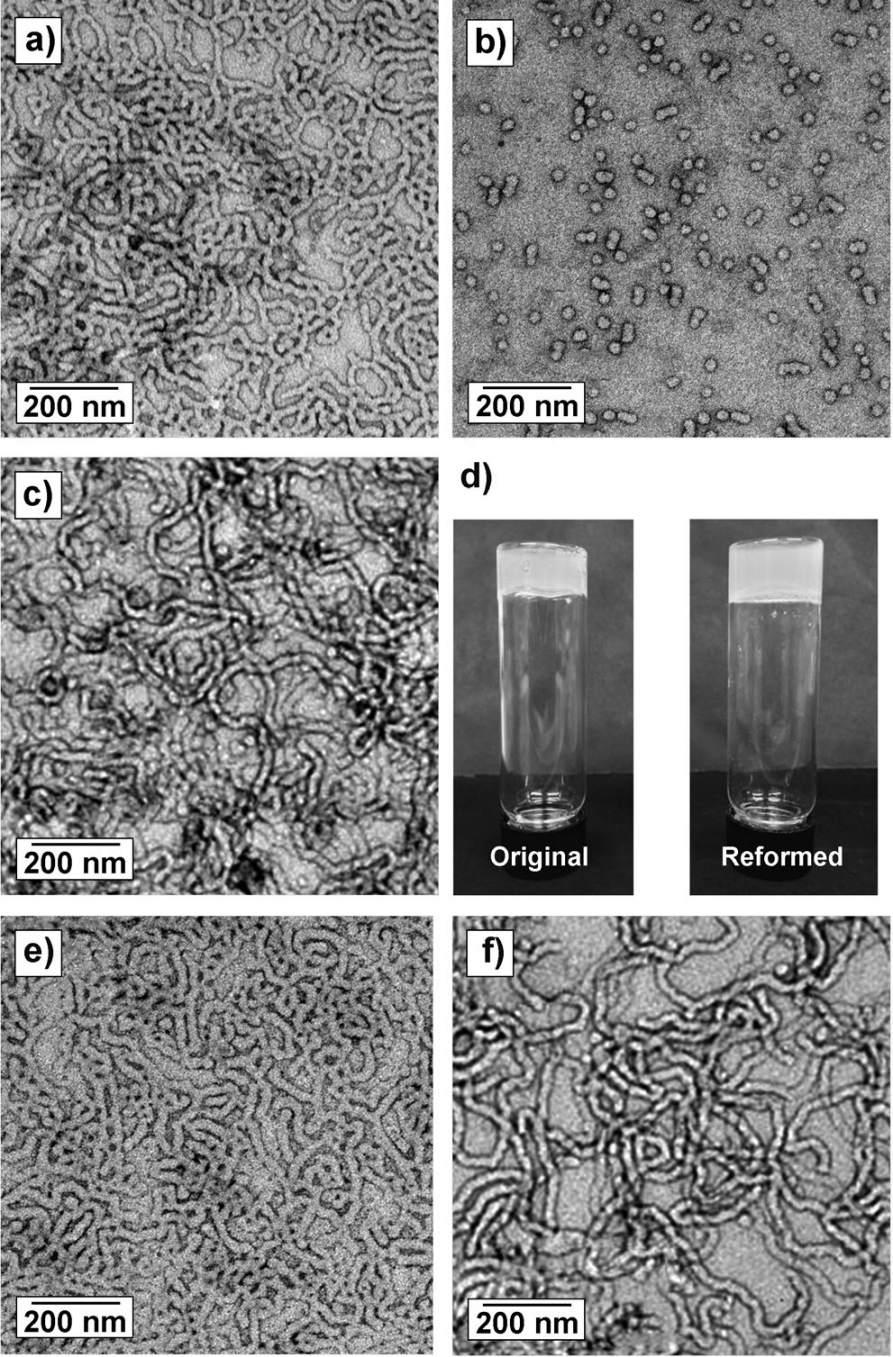
TEM images obtained on addition of NaOH followed by dilution of a 10 % w/w aqueous dispersion of a HOOC–PGMA_56_–PHPMA_155_ diblock copolymer prepared using the carboxylic acid functionalized PETTC RAFT agent: a) pH 3.5 (initial worms); b) pH 6.0 (spheres); c) pH 3.5 (reformed worms). d) Photographs of the transparent free-standing gels formed by the worm phase corresponding to (a) and (c). Control experiments: TEM images obtained for a H_3_COOC–PGMA_59_–PHPMA_160_ diblock copolymer prepared using a methylated PETTC RAFT agent (Me-PETTC) at: e) pH 3.5 and f) pH 6.0, both containing worms. No worm-to-sphere transition is detected in the absence of carboxylic acid end-groups (e) and (f).

Acid titration studies of the HOOC–PGMA_56_ macro-CTA in aqueous solution (see Figure S1 in the Supporting Information) indicated that the p*K*_a_ of the terminal carboxylic acid group is approximately 4.7. Thus we hypothesized that ionization of the carboxylic acid end-group introduced by the PETTC RAFT agent was the most likely cause of the pH-responsive behavior exhibited by the HOOC–PGMA_56_–PHPMA_155_ diblock copolymer. To examine this hypothesis, control experiments were performed using a methylated PETTC RAFT agent (Me-PETTC) to prepare a PGMA macro-CTA with a mean degree of polymerization (DP) of 59, which was subsequently chain-extended with HPMA to produce an analogous near-monodisperse H_3_COOC–PGMA_59_–PHPMA_160_ diblock copolymer (see Figure S2 in the Supporting Information).

As expected, only thermoresponsive gelation was observed for this copolymer; TEM studies confirmed that the original copolymer worm morphology remained intact at 20 °C, regardless of the solution pH (see Figure 2 e and f). In a further series of experiments, dynamic light scattering (DLS) and zeta potential studies were conducted as a function of solution pH for copolymer worms prepared using either the PETTC or Me-PETTC RAFT agents, respectively (Figure 3).

**Figure 3:**
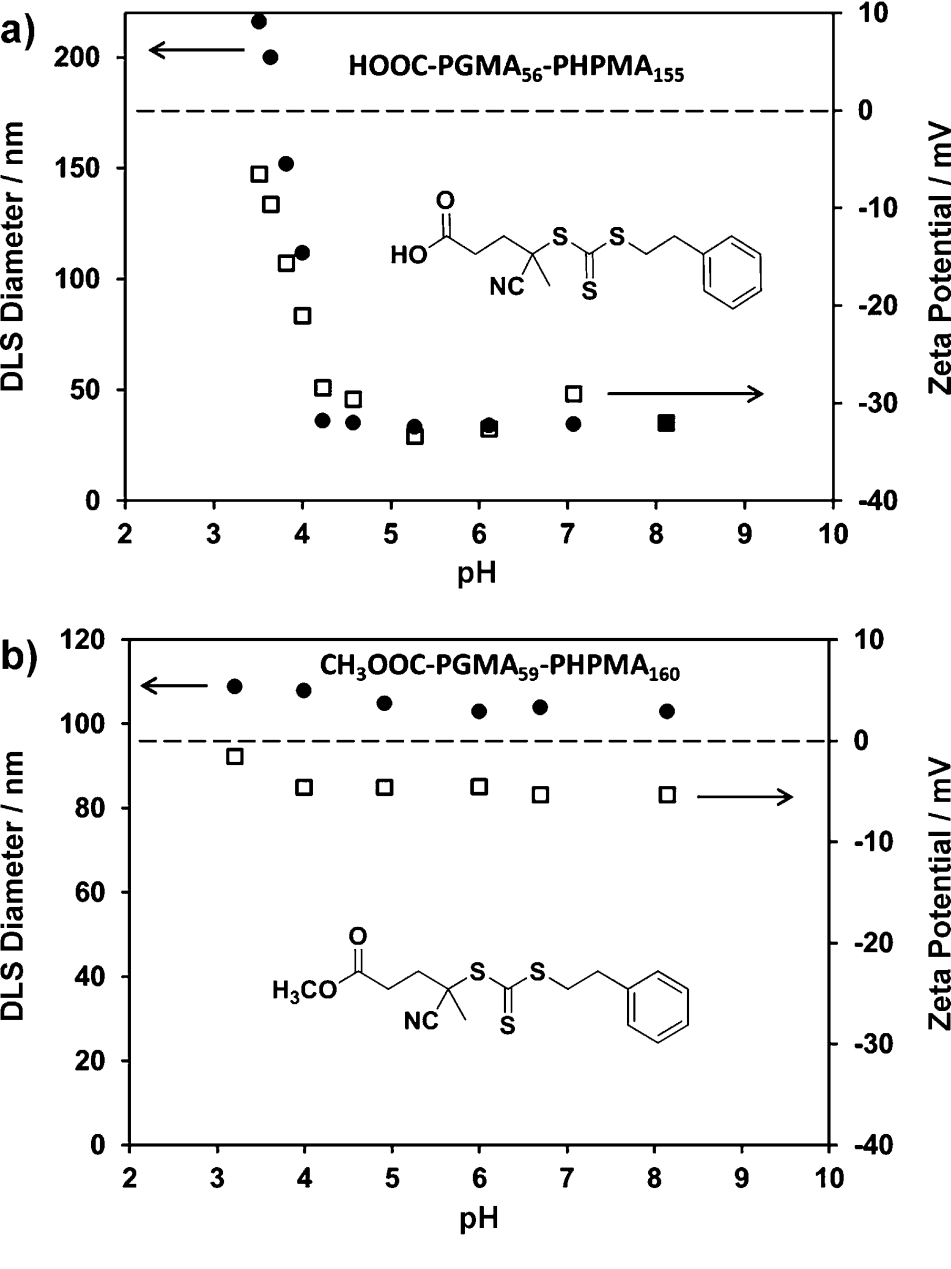
Variation of hydrodynamic particle diameter[10] (filled circles) and zeta potential (open squares) with solution pH values recorded for a 0.1 % w/w aqueous dispersions of a) HOOC–PGMA_56_–PHPMA_155_ pH-responsive worms and b) H_3_COOC–PGMA_59_–PHPMA_160_ pHinsensitive worms.

Examining the HOOC–PGMA_56_–PHPMA_155_ copolymer by DLS, the significant reduction in its apparent particle dimensions[10] from 220 nm to 40 nm that is observed on increasing the solution pH value from pH 3.5 to pH 7.0 provides good evidence for a worm-to-sphere transition (see Figure 3 a). This morphological transition was confirmed by TEM studies of the dried diluted aqueous dispersions (see Figure 2 a and 2 b). Moreover, the critical pH value for the worm-to-sphere transition appears to be close to the p*K*_a_ of the terminal carboxylic acid. It is also emphasized that ionization of this end-group leads to significantly greater anionic character for the nano-objects (from −5 mV for the original worms at pH 3.5 to around −30 mV for the spheres at pH 5–8). Thus ionization of a single carboxylic acid group at the end of each PGMA–PHPMA chain increases the degree of hydration of the stabilizer block sufficiently to lower the packing parameter, *P*, from worms (0.33<*P*<0.50) to spheres (*P*<0.33),[4a,4c] hence inducing the morphological transition. This subtle end-group effect serves to illustrate the rather delicate hydrophilic–hydrophobic balance (or relatively narrow *P* range) that is required for formation of the copolymer worm morphology. Further evidence to support this end-group ionization effect was obtained by examining the effect of added salt. Thus, a gel composed of HOOC–PGMA_56_–PHPMA_155_ copolymer worms synthesized in the presence of 100 mM KCl at pH 3.4 did not change in morphology on switching the solution pH to pH 7.5, as judged by the tube inversion test (see Figure S3 in the Supporting Information). Aqueous electrophoresis and DLS studies indicate the presence of weakly anionic worms (apparent diameter=212 nm; zeta potential≈ -5.7 mV). Thus, added salt screens the anionic charge arising from ionization of the terminal carboxylic acid groups, so the worm- to-sphere transition is not detected under these conditions.

In contrast, DLS and aqueous electrophoresis studies of the analogous H_3_COOC–PGMA_59_–PHPMA_160_ worms prepared using the Me-PETTC RAFT agent over the same pH range confirms that there is barely any discernible change in either particle size or zeta potential (see Figure 3 b). This indicates that these worms are pH-insensitive as they contain no terminal ionizable COOH group. Furthermore, we detect similar pH-responsive behavior for PGMA–PHPMA copolymer worms prepared using other carboxylic acid functionalized RAFT agents, such as 4-cyanopentanoic acid dithiobenzoate (data not shown).

Gel rheology studies were also performed as a function of pH on 10 % w/w HOOC–PGMA_56_–PHPMA_155_ (Figure 4, open and filled circles) and H_3_COOC–PGMA_59_–PHPMA_160_ (open and filled squares) diblock copolymer dispersions at 25 °C. At around pH 3.7, both copolymers formed soft, free-standing worm gels, with *G*′ values of around 10^2^ Pa. These gel strengths are comparable those reported by Blanazs and co-workers for a closely related gel composed of PGMA_54_–PHPMA_140_ copolymer worms at neutral pH.[9] Increasing the solution pH to 4.8 or above led to a dramatic reduction in *G*′ values for the HOOC–PGMA_56_–PHPMA_155_ diblock copolymer, with concomitant transformation of the gel into a free-flowing liquid (filled circles). On returning to the original pH, regelation occurred and a *G*′ value comparable to the original value was obtained (open circles). In marked contrast, the value of *G*′ for the H_3_COOC–PGMA_59_–PHPMA_160_ diblock copolymer remained essentially constant from pH 3.4 to 7.5 (squares). Furthermore, this pH-insensitive worm gel exhibited thermoresponsive behavior from pH 3.4 to pH 7.5, while the HOOC–PGMA_56_–HPMA_155_ gel was only thermoresponsive (as judged by tube inversion tests) to below pH 4.7, which corresponds to the p*K*_a_ of the terminal carboxylic acid. Thus, these gel rheology observations made at 10 % w/w copolymer concentration are fully consistent with our TEM, DLS, and aqueous electrophoresis studies of highly dilute copolymer dispersions and further support our contention that end-group ionization alone can be sufficient for non-ionic diblock copolymer nano-objects to exhibit a reversible worm-to-sphere transition. Furthermore, we have exploited this new physical insight to design analogous pH-sensitive vesicles based on non-ionic HOOC–PGMA_43_–PHPM_200_ diblock copolymers (Figure 5). In this case, addition of NaOH to a free-flowing aqueous dispersion of vesicles leads to the gradual formation of a free-standing gel over a timescale of 8–10 hours at 20 °C. Subsequent TEM studies of the diluted gel phase confirmed a morphology switch from vesicles to worms. However, in this case the transition is not reversible: addition of acid produces a solid white paste, rather than the original turbid vesicular dispersion.

**Figure 4:**
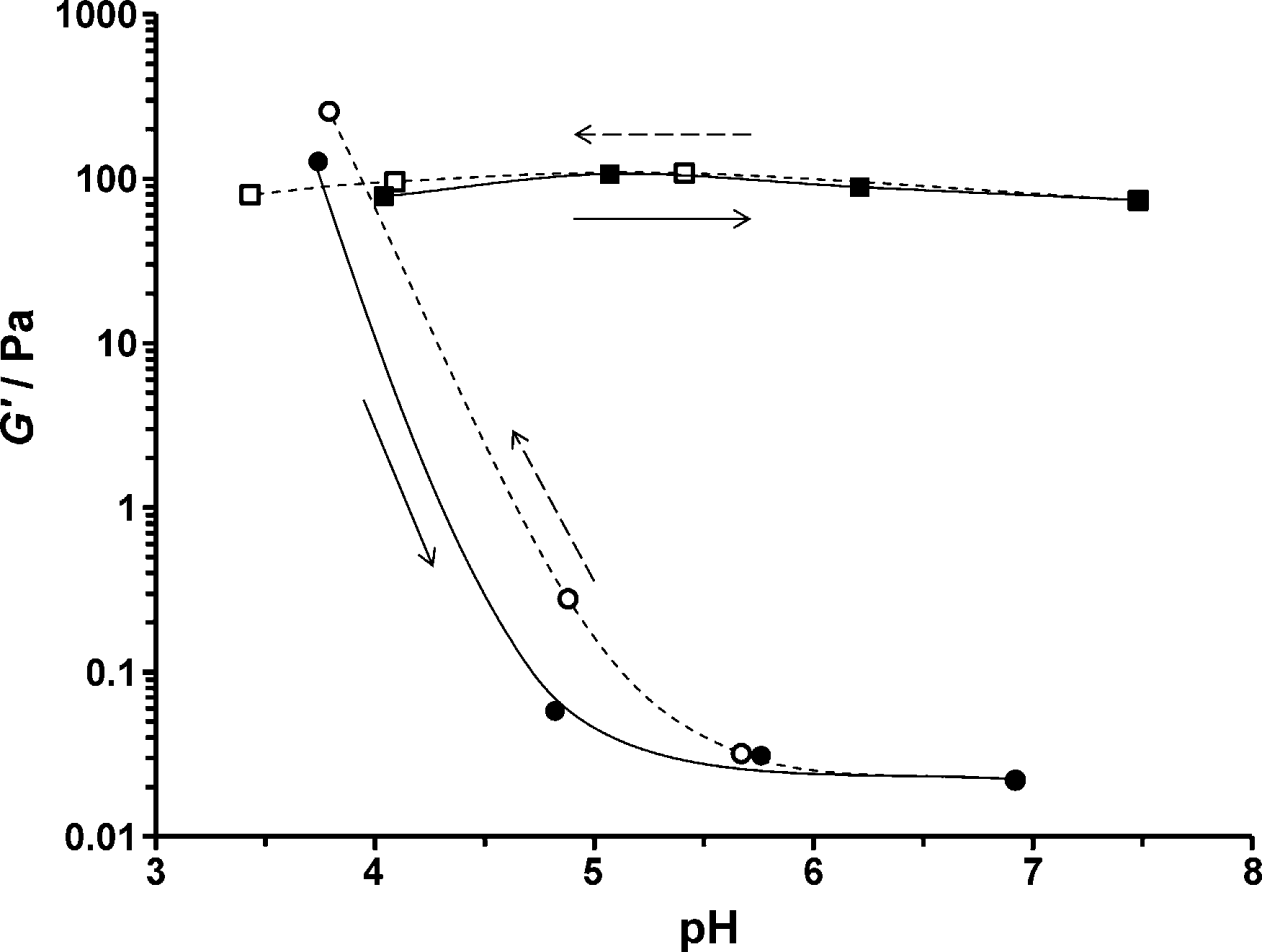
Variation in gel storage modulus (*G*′) for a 10 % w/w HOOC–PGMA_56_–PHPMA_155_ diblock copolymer worm/sphere dispersion at 25 °C during a pH switch from 3.7 to 6.9 (filled circles), followed by a switch back to pH 3.8 (open circles). As a control experiment, a gel composed of H_3_COOC–PGMA_59_–PHPMA_160_ diblock copolymer worms showed essentially no change in the storage modulus, *G*′, from pH 3.4 to 7.5 (open and filled squares). The solid lines represent increasing pH, while the dotted lines represent decreasing pH.

**Figure 5:**
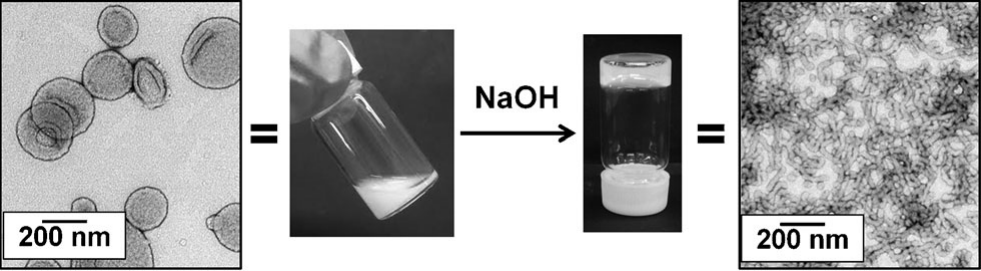
Representative TEM images obtained before (left) and after (right) addition of NaOH to a 10 % w/w aqueous dispersion of HOOC–PGMA_43_–PHPMA_200_ diblock copolymer nanoparticles, followed by dilution. Photographs of the two dispersions indicate their differing physical forms: free-flowing turbid vesicular dispersion (left) and free-standing gel composed of copolymer worms (right).

In summary, we demonstrate that non-ionic diblock copolymers can unexpectedly exhibit pH-responsive behavior. More specifically, gels composed of PGMA–PHPMA copolymer worms are converted into free-flowing dispersions comprising spheres on increasing the solution pH. This pH-responsive behavior is reversible and is driven by ionization of a single terminal carboxylic acid end-group on each PGMA stabilizer block, which serves to illustrate the remarkably subtle nature of the worm-to-sphere order–order transition. Moreover, it is emphasized that such pH-responsive diblock copolymer nano-objects differ from conventional pH-responsive weak polyelectrolytes since only minimal amounts of base (or acid) are required to induce the change in copolymer morphology. This may be important if repeated pH cycling is required, as the otherwise problematic accumulation of background salt is minimized.[11] It is also shown that a pH switch can induce an irreversible vesicle-to-worm morphological transition. This work represents an important new paradigm for pH-induced morphological transitions exhibited by block copolymer nano-objects.
