# Supplementary material for: pH-Responsive Non-Ionic Diblock Copolymers: Ionization of Carboxylic Acid End-Groups Induces an Order–Order Morphological Transition
Source: Angew Chem Int Ed Engl. 2014 Nov 21;54(4):1279–83. doi: 10.1002/anie.201409799 (PMC4312895; doi:10.1002/anie.201409799)
Supplement: Supplementary file 1 [file anie0054-1279-sd1.pdf]

Angewandte Chemie
Eine Zeitschrift der Gesellschaft Deutscher Chemiker

Supporting Information
© Wiley-VCH 2015
69451 Weinheim, Germany

pH-Responsive Non-Ionic Diblock Copolymers: Ionization of Carboxylic Acid End-Groups Induces an Order–Order Morphological Transition**

*Joseph R. Lovett, Nicholas J. Warren, Liam P. D. Ratcliffe, Marzena K. Kocik, and Steven P. Armes**

anie_201409799_sm_miscellaneous_information.pdf

Experimental Section

Materials

Glycerol monomethacrylate (GMA; 99.8%) was donated by GEO Specialty Chemicals (Hythe, UK) and used without further purification. 2-Hydroxypropyl methacrylate (HPMA), 4,4'-azobis(4-cyanopentanoic acid) (ACVA; V-501; 99%), dicyclohexylcarbodiimide (DCC), 4-(dimethylamino)pyridine (DMAP), ethanol (99%, anhydrous grade), ethyl acetate, petroleum ether, methanol and dichloromethane were purchased from Sigma-Aldrich UK and were used as received. All solvents were of HPLC quality and were purchased from Fisher Scientific (Loughborough, UK). 4-Cyano-4-(2-phenylethanesulfanylthiocarbonyl)-sulfanylpentanoic acid (PETTC) was prepared and purified as reported elsewhere (see M. Semsarilar et al., *Macromolecules*, **2012**, *45*, 5091-5098).

Synthesis of poly(glycerol monomethacrylate) (HOOC-PGMA) macro-CTA

GMA (10.0 g, 62.4 mmol), PETTC RAFT agent (0.303 g, 0.89.2 mmol; target DP = 70), and ACVA (0.050 g, 0.18 mmol; PETTC/ACVA molar ratio = 5.0) were accurately weighed into a 100 ml round-bottomed flask. Anhydrous ethanol (previously purged with nitrogen for 1 h) was added to produce a 40% w/w solution, which was placed in an ice bath and purged under nitrogen for 45 min. at 0 °C. The sealed flask was immersed in an oil bath set at 70 °C and stirred for 2 h. The polymerization was then quenched at approximately 78% conversion by exposure to air, followed by cooling to room temperature. Methanol (10 ml) was added to dilute the reaction solution, followed by precipitation into a ten-fold excess of dichloromethane in order to remove unreacted GMA monomer. The precipitate was isolated via filtration and washed with excess dichloromethane before being dissolved in methanol (40 ml). The crude polymer was precipitated for a second time by addition to excess dichloromethane and isolated via filtration. It was then dissolved in water and freeze-dried overnight to afford a yellow solid. ^{1}H NMR studies indicated a mean degree of polymerization of 56 via end-group analysis. DMF GPC studies (refractive index detector; calibrated against a series of near-monodisperse poly(methyl methacrylate) standards) indicated an M_n of 14,100 and an M_w/M_n of 1.17.

Synthesis of HOOC-PGMA$_{56}$-PHPMA$_{155}$ diblock copolymer worms via RAFT aqueous dispersion polymerization

A typical protocol for the chain extension of HOOC-PGMA$_{56}$ macro-CTA with 155 units of HPMA via RAFT aqueous dispersion polymerization is as follows: PGMA$_{56}$ macro-CTA (0.208 g, 0.022 mmol), HPMA monomer (0.50 g, 3.4 mmol) ACVA (2.1 mg, 0.006 mmol; PGMA$_{56}$ macro-CTA: ACVA molar ratio = 3.0) were added to a 25 ml round-bottomed flask, prior to addition of water to produce a 10% w/w solution. The reaction solution was purged under nitrogen for 30 min at 20 °C prior to immersion into an oil bath set at 70 °C. The reaction mixture was stirred for 4 h to ensure almost complete conversion of the HPMA monomer (> 99 % by ^{1}H NMR analysis) and was quenched by exposure to air, following by cooling to ambient temperature. The resulting worm gel was characterized by DLS, TEM and rheology without further purification.

Methylation of PETTC chain transfer agent

PETTC (0.20 g, 0.59 mmol) was dissolved in anhydrous dichloromethane (1.50 ml) in a 10 ml round-bottomed flask, which was cooled in an ice bath to 0 °C. DMAP (0.014 g, 0.12 mmol) and excess anhydrous methanol (0.10 g) were added to the stirred solution at 0 °C. DCC (0.14 g, 0.66 mmol) was added gradually over 5 min. This reaction mixture was allowed to warm up to 20 °C and stirred continuously for 16 h prior to filtration to remove the insoluble side-product (dicyclohexyl urea). The filtrate was then washed twice with acidic water (pH 3) and de-ionized water (pH 6) before being dried over magnesium sulfate. Finally, dichloromethane was removed under vacuum to produce an orange oil. ^{1}H NMR spectroscopy and mass spectrometry analysis confirmed the chemical structure expected for Me-PETTC.

Synthesis of poly(glycerol monomethacrylate) (H_3COOC-PGMA) macro-CTA

GMA (4.00 g, 25.0 mmol), Me-PETTC RAFT agent (0.126 g, 0.36 mmol; target DP = 70), and ACVA (0.020 g, 0.070 mmol; Me-PETTC/ACVA molar ratio = 5.0) were accurately weighed into a 25 ml round-bottomed flask. Anhydrous ethanol (previously purged with nitrogen for 1 h) was added to produce a 40% w/w solution, which was placed in an ice bath and purged under nitrogen for 45 min. at 0 °C. The sealed flask was immersed in an oil bath set at 70 °C and stirred for 2 h. The polymerization was then quenched at approximately 72 % conversion by exposure to air, followed by cooling to room temperature. Methanol (5.0 ml) was added to dilute the reaction solution, followed by precipitation into a ten-fold excess of dichloromethane in order to remove unreacted GMA monomer. The precipitate was isolated via filtration and washed with excess dichloromethane before being dissolved in methanol (20 ml). The crude polymer was precipitated for a second time by addition to excess dichloromethane and isolated via filtration. It was then dissolved in water and freeze-dried overnight to afford a yellow solid. ^{1}H NMR studies indicated a mean degree of polymerization of 59 via end-group analysis. DMF GPC studies (refractive index detector; calibrated against a series of near-monodisperse poly(methyl methacrylate) standards) indicated an M_n of 15,600 and an M_w/M_n of 1.20.

Synthesis of H_3COOC-PGMA$_{59}$-PHPMA$_{160}$ diblock copolymer worms via RAFT aqueous dispersion polymerization

A typical protocol for the chain extension of H_3COOC-PGMA$_{56}$ macro-CTA with 160 units of HPMA via RAFT aqueous dispersion polymerization is as follows: PGMA$_{59}$ macro-CTA (0.167 g, 0.017 mmol), HPMA monomer (0.40 g, 2.8 mmol) ACVA (1.6 mg, 0.006 mmol; PGMA$_{56}$ macro-CTA: ACVA molar ratio = 3.0) were added to a 25 ml round-bottomed flask, prior to addition of water to produce a 10% w/w solution. The reaction solution was purged under nitrogen for 30 min at 20 °C prior to immersion into an oil bath set at 70 °C. The reaction mixture was stirred for 4 h to ensure almost complete conversion of the HPMA monomer (> 99 % by ^{1}H NMR analysis) and was quenched by exposure to air, following by cooling to ambient temperature. The resulting worm gel was characterized by DLS, TEM and rheology without further purification.

Instrumentation

NMR spectroscopy. ^{1}H NMR spectra were recorded using a 500 MHz Bruker Avance-500 spectrometer (64 scans averaged per spectrum).

Gel Permeation Chromatography (GPC). Polymer molecular weights and polydispersities were determined using a DMF GPC set-up operating at 60 °C and comprising two Polymer Laboratories PL gel 5 μm Mixed-C columns connected in series to a Varian 390-LC multi-detector suite (refractive index detector) and a Varian 290-LC pump injection module. The GPC eluent was HPLC-grade DMF containing 10 mM LiBr at a flow rate of 1.0 mL min^{-1}. DMSO was used as a flow-rate marker. Calibration was conducted using a series of ten near-monodisperse poly(methyl methacrylate) standards (M_n = 625 to 2,480,000 g mol^{-1}). Chromatograms were analyzed using Varian Cirrus GPC software (version 3.3).

Dynamic Light Scattering. DLS studies were conducted using a Malvern Zetasizer NanoZS instrument at 25 ^{o}C. Studies were performed on 0.10% w/w aqueous dispersions in disposable cuvettes at a fixed scattering angle of 173°. Intensity-average hydrodynamic diameters were calculated via the Stokes-Einstein equation using a non-negative least-squares (NNLS) algorithm. All data were averaged over three consecutive runs.

Transmission Electron Microscopy (TEM). Solutions were diluted 100-fold at 20 °C to generate 0.10% w/w dispersions. Images obtained at lower pH were prepared by diluting solutions in acidic water. Copper/palladium TEM grids (Agar Scientific) were surface-coated in-house to yield a thin film of amorphous carbon. The grids were then plasma glow-discharged for 30 seconds to create a hydrophilic surface. Individual samples (0.10% w/w, 12 μL) were adsorbed onto the freshly glow-discharged grids for one minute and then blotted with filter paper to remove excess solution. To stain the aggregates, uranyl formate (0.75% w/w) solution (9 μL) was soaked on the sample-loaded grid for 20 s and then carefully blotted to remove excess stain. The grids were then dried using a vacuum hose. Imaging was performed on a Phillips CM100 instrument at 100 kV, equipped with a Gatan 1 k CCD camera.

Rheology Studies. Storage moduli (G') were determined for the HOOC-$PGMA_{56}$-$HPMA_{155}$ and CH_3OOC-$PGMA_{59}$-$PHPMA_{160}$ diblock copolymer worm gel at 25 °C using a TA Instruments AR-G2 rheometer with a fixed strain of 1.0 % at an angular frequency of 1.0 rad s^{-1}. A cone-and-plate geometry (40 mm 2° aluminum cone) was used for these measurements.

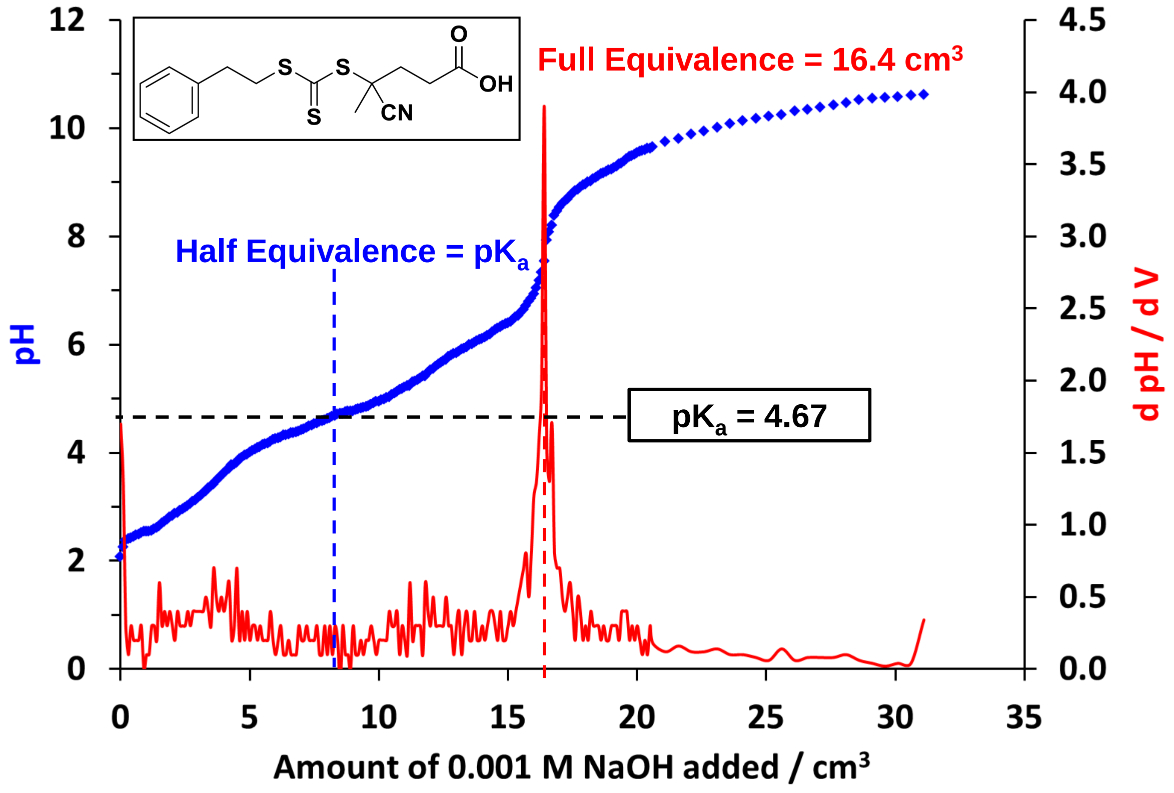

Figure S1. Acid titration curve obtained for the $PGMA_{56}$ macro-CTA used in this work. The pK_a of 4.67 is consistent with that expected for an isolated carboxylic acid end-group.

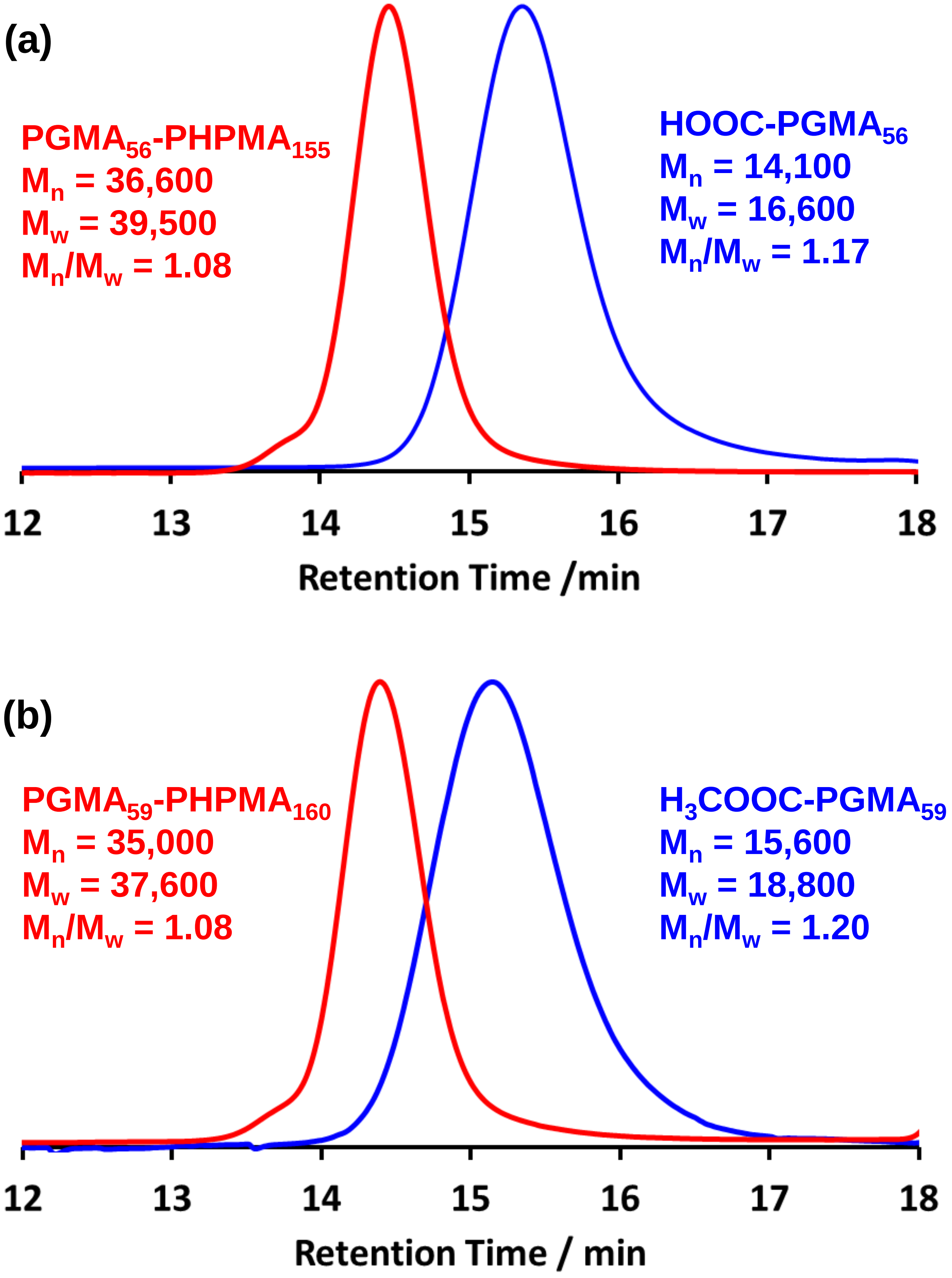

Figure S2. DMF gel permeation chromatographs obtained for (a) HOOC-$PGMA_{56}$ macro-CTA and the corresponding HOOC-$PGMA_{56}$-$PHPMA_{160}$ diblock copolymer and (b) H_3COOC-$PGMA_{59}$ macro-CTA and corresponding H_3COOC-$PGMA_{59}$-$PHPMA_{160}$ diblock copolymer. In both cases high blocking efficiencies and low final copolymer polydispersities were obtained.

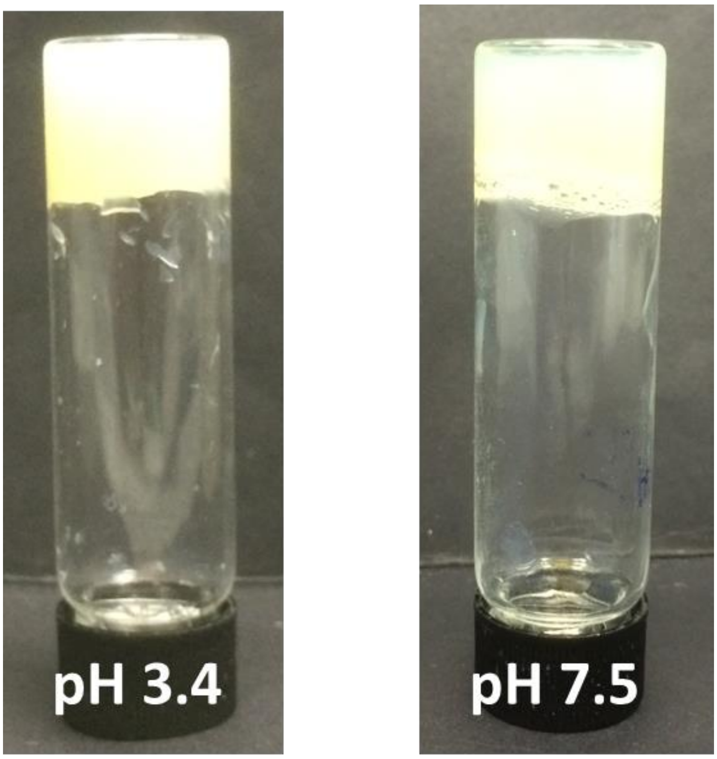

Figure S3. Digital images obtained for A HOOC-$PGMA_{56}$-$PHPMA_{155}$ worm gel synthesized in the presence of 100 Mm KCl at an initial pH of 3.4 (left-hand image) and after a pH switch to 7.5 (right-hand image). The tube inversion test confirms that this sample remains a free-standing gel on switching the pH, which indicates that no worm-to-sphere transition occurs in the presence of sufficient added salt.
